# Dataset of Speech Production in intracranial Electroencephalography

**DOI:** 10.1038/s41597-022-01542-9

**Published:** 2022-07-22

**Authors:** Maxime Verwoert, Maarten C. Ottenhoff, Sophocles Goulis, Albert J. Colon, Louis Wagner, Simon Tousseyn, Johannes P. van Dijk, Pieter L. Kubben, Christian Herff

**Affiliations:** 1 Department of Neurosurgery, School for Mental Health and Neuroscience, Maastricht University, Maastricht, The Netherlands; 2 Academic Center for Epileptology, Kempenhaeghe/Maastricht University Medical Center, Heeze, The Netherlands; 3 Department of Orthodontics, Ulm University, Ulm, Germany; 4 Department of Electrical Engineering, Eindhoven University of Technology, Eindhoven, The Netherlands; 5 Academic Center for Epileptology, Kempenhaeghe/Maastricht University Medical Center, Maastricht, The Netherlands

**Keywords:** Neural decoding, Brain-machine interface, Language

## Abstract

Speech production is an intricate process involving a large number of muscles and cognitive processes. The neural processes underlying speech production are not completely understood. As speech is a uniquely human ability, it can not be investigated in animal models. High-fidelity human data can only be obtained in clinical settings and is therefore not easily available to all researchers. Here, we provide a dataset of 10 participants reading out individual words while we measured intracranial EEG from a total of 1103 electrodes. The data, with its high temporal resolution and coverage of a large variety of cortical and sub-cortical brain regions, can help in understanding the speech production process better. Simultaneously, the data can be used to test speech decoding and synthesis approaches from neural data to develop speech Brain-Computer Interfaces and speech neuroprostheses.

## Abstract (summary)

**Table:** 

Measurement(s)	Brain activity
Technology Type(s)	Stereotactic electroencephalography
Sample Characteristic - Organism	Homo sapiens
Sample Characteristic - Environment	Epilepsy monitoring center
Sample Characteristic - Location	The Netherlands

## Background & Summary

Brain-Computer Interfaces (BCIs)^1^ ﻿that directly decode speech from neural activity have recently gained large attention as they could provide an intuitive means of communication for patients who lost the ability to speak^2–7^.

The creation of a speech neuroprosthesis depends on a firm understanding of the speech production process in the brain, the particular timing of brain regions involved and where to best decode them. Despite a number of existing models on the speech production process^8,9^, the precise role of all areas involved has yet to be understood. Recent advances highlight that deeper brain structures, such as the hippocampus^10–12^ and thalamus^13,14^, are also involved in language in general and speech production specifically. A dataset providing accessible data for a simple speech production task in cortical and deeper brain structures could help to further understand this intricate process. This understanding may additionally aid functional language mapping prior to resective surgery in patients suffering from pharmaco-resistant epilepsy^15^.

Despite the fact that a full understanding of speech production is currently lacking, great advances have been made in the field of speech neuroprostheses recently. The decoding of a textual representation by decoding phonemes^16,17^, phonetic^18^ or articulatory^19^ features, words^20^, full sentences^21–24^ or spotting of speech keywords^25^ is possible from neural recordings during actual speech production. Results are becoming robust enough for first trials in speech impaired patients^7^. To facilitate more natural communication, some studies aimed at directly synthesizing an audio waveform of speech from neural data recorded during speech production^26–29^. Initial results indicate that the decoding of speech processes is possible from imagined speech production from offline data^30–32^ and in real-time^33,34^.

Most of these recent advances employ electrocorticography (ECoG), an invasive recording modality of neural activity that provides high temporal and spatial resolution and high signal-to-noise ratio^35^. Additionally, ECoG is less affected by movement artifacts than non-invasive measures of neural activity. Other studies have used intracortical microarrays to decode speech^36–39^ or a neurotrophic electrode^40^ to synthesize formant frequencies^41,42^ from the motor cortex. An alternative measure of intracranial neural activity is stereotactic EEG (sEEG), in which electrode shafts are implanted into the brain through small burr holes^43^. sEEG is considered to be minimally invasive, as a large craniotomy is not necessary and the infection risk is therefore smaller^44^. Additionally, the method of implanting the electrodes is very similar to that used in Deep Brain Stimulation (DBS), a method that has been used in the treatment of Parkinson’s Disease for several decades. In DBS, electrodes routinely remain implanted for many years, giving hope for the potential of sEEG for long-term BCIs.

Similar to ECoG, sEEG is used in the monitoring of epilogenic zones in the treatment of refractory epilepsy. Between 5 and 15 electrode shafts are typically implanted covering a large variety of cortical and sub-cortical brain areas. Here lies one of the main differences to ECoG: instead of high density coverage of specific regions, sEEG provides sparse sampling of multiple regions. This sparse sampling could provide great potential for various BCI applications as most of them involve processes in deep (sub-cortical or within sulci) and spatially disparate, bilateral, brain regions^45^. For example, besides the primary motor cortex, movement can also be decoded well from the basal ganglia^46^ and supramarginal gyrus^47^, amongst others. With sEEG, these areas can be recorded simultaneously, leveraging multiple sources of potential information.

Invasive recordings of neural activity are usually obtained during a seizure localization procedure or glioma resection surgery and are therefore not available to many researchers working on traditional speech decoding or speech synthesis technologies. These researchers are part of an active research community investigating the potential of non-invasive brain measurement technologies for speech neuroprostheses. Techniques include scalp-electroencephalography (EEG)^48–54^, providing high temporal resolution, especially the Kara One database^55^ provides the foundation for many studies; Magnetoencephalography^56,57^, providing more localized information than EEG due to a larger amount of sensors; and Functional Near Infrared Spectroscopy^58–61^, providing localized information of cortical hemoglobin levels. The advances made by this community could also benefit invasive speech neuroprostheses, a dataset that is provided to everyone could be used to evaluate and leverage their approaches.

**Fig. 1:**
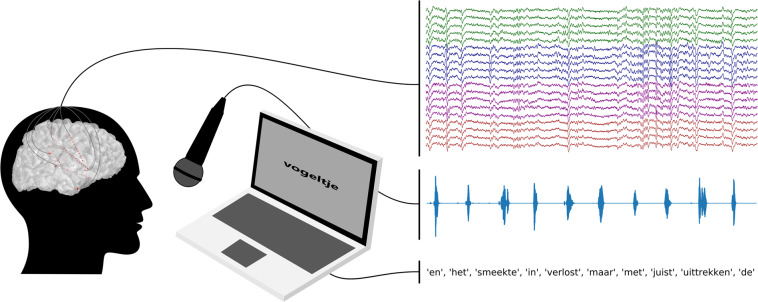
Intracranial EEG and acoustic data are recorded simultaneously while participants read Dutch words shown on a laptop screen. Traces on the right of the figure represent 30 seconds of iEEG, audio and stimulus data. The colors in the iEEG traces represent different electrode shafts.

To facilitate an increased understanding of the speech production process in the brain, including deeper brain structures, and to accelerate the development of speech neuroprostheses, we provide this dataset of 10 participants speaking prompted words aloud while audio and intracranial EEG data are recorded simultaneously (Fig. 1).

## Methods

### Participants

A total of 10 participants suffering from pharmaco-resistant epilepsy participated in our experiment (mean age 32 years (range 16–50 years); 5 male, 5 female). Participants were implanted with sEEG electrodes (Table 1) as part of the clinical therapy for their epilepsy. Electrode locations were purely determined based on clinical necessity. All participants joined the study on a voluntary basis and gave written informed consent. Experiment design and data recording were approved by the Institutional Review Boards of both Maastricht Unviversity and Epilepsy Center Kempenhaeghe. Data recording was conducted under the supervision of experienced healthcare staff. All participants were native speakers of Dutch. Participants’ voices were pitch-shifted with a randomized offset between 1 and 3 semitones up or down that was constant over the entire recording to ensure anonymity.

**Table 1:** Number of implanted and recorded electrodes of each participant.

	sub-01	sub-02	sub-03	sub-04	sub-05	sub-06	sub-07	sub-08	sub-09	sub-10	Total
Implanted	133	234	184	117	61	155	134	56	119	124	1317
Recorded	127	127	127	115	60	127	127	54	117	122	1103

Note that the recorded number does not include the reference electrode, which is inherently carried in all recorded electrodes.

### Experimental design

In this study, participants were asked to read aloud words that were shown to them on a laptop screen (Fig. 1). One random word from the stimulus library (the Dutch IFA corpus^62^ extended with the numbers one to ten in word form) was presented on the screen for a duration of 2 seconds during which the participant read the word aloud once. This relatively large window accounts for differences in word length and pronunciation speed. After the word, a fixation cross was displayed for 1 second. This was repeated for a total of 100 words, resulting in a total recording time of 300 seconds for each participant. The presented stimuli and timings were saved for later processing, hereafter referred to as stimulus data.

### Data acquisition

Participants were implanted with platinum-iridium sEEG electrode shafts (Microdeep intracerebral electrodes; Dixi Medical, Beçanson, France) with a diameter of 0.8 mm, a contact length of 2 mm and a inter-contact distance of 1.5 mm. Each electrode shaft contained between 5 and 18 electrode contacts.

Neural data was recorded using two or more Micromed SD LTM amplifier(s) (Micromed S.p.A., Treviso, Italy) with 64 channels each. Electrode contacts were referenced to a common white matter contact. Data were recorded at either 1024 Hz or 2048 Hz and subsequently downsampled to 1024 Hz. We used the onboard microphone of the recording notebook (HP Probook) to record audio at 48 kHz. Audio data was subsequently pitch-shifted to ensure our participants’ anonymity using LibRosa^63^. We used LabStreamingLayer^64^ to synchronize the neural, audio and stimulus data.

### Anatomical labeling

**Fig. 2:**
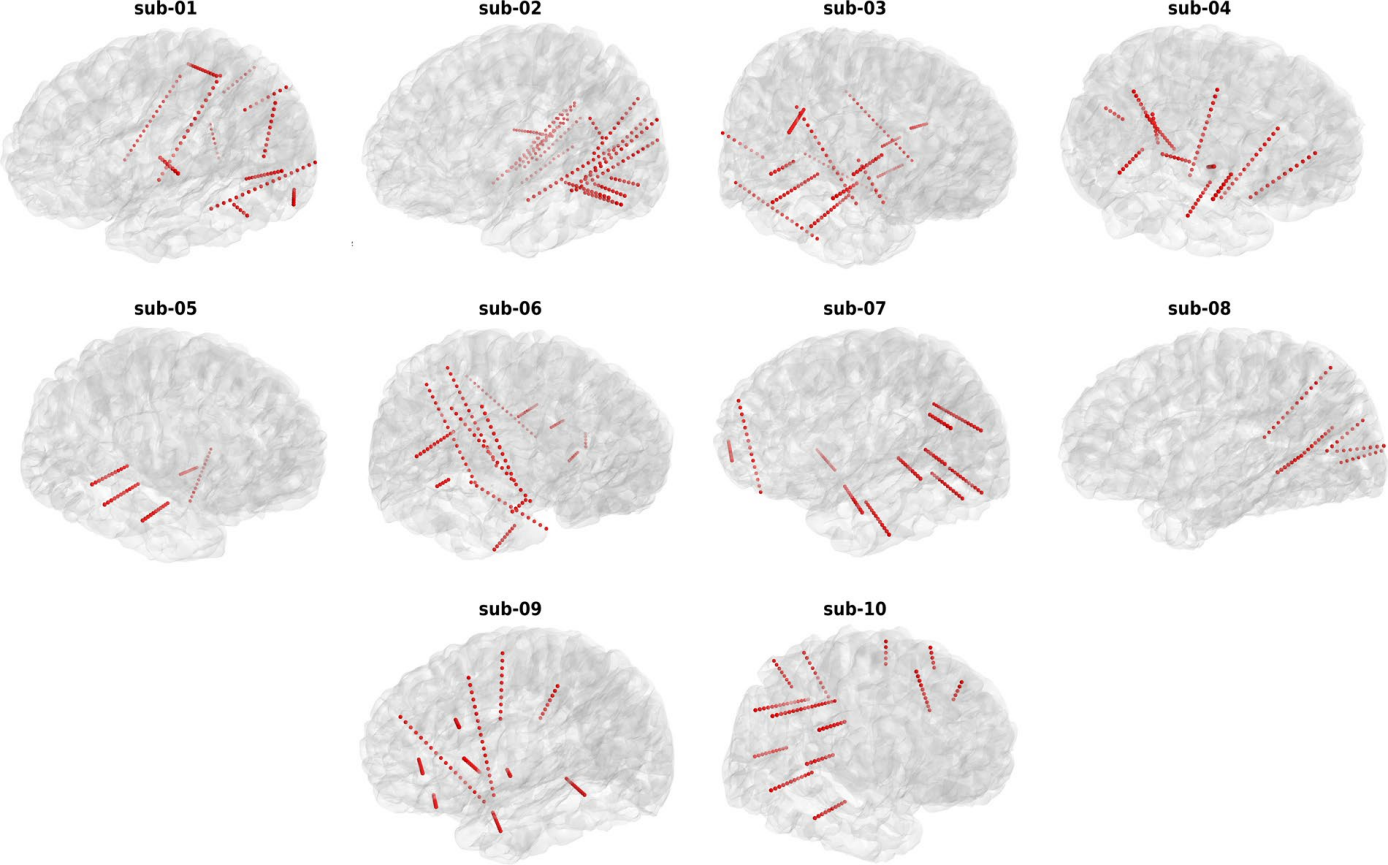
Electrode locations of each participant in the surface reconstruction of their native anatomical MRI. Each red sphere represents an implanted electrode channel.

Electrode locations (Fig. 2) were detected using the img_pipe Python package^65^ for anatomical labeling of intracranial electrodes. Within the package, for each participant, a pre-implantation anatomical T1-weighted Magnetic Resonance Imaging (MRI) scan was parcellated using Freesurfer (http://surfer.nmr.mgh.harvard.edu/), a post-implantation Computer Tomography (CT) scan was co-registered to the MRI scan and electrode contacts were manually localized. The anatomical location label of each contact was automatically extracted from the Destrieux atlas^66^ based parcellation.

**Fig. 3:**
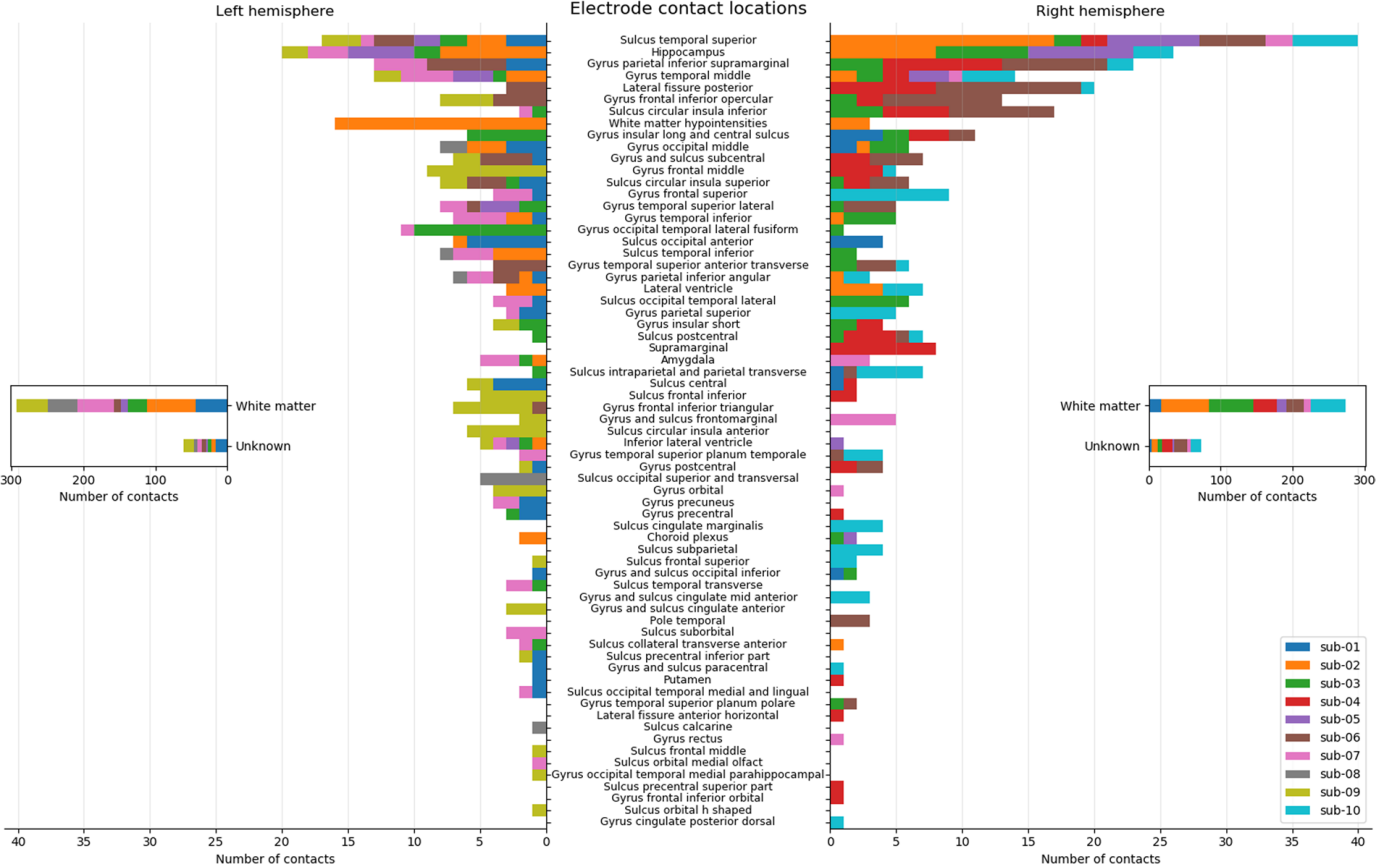
Number of electrode contacts in cortical and subcortical areas across all participants. Colors indicate participants. Lengths of the bars show the number of electrodes in the specified region. Note the deviant x-axis for the white matter and unknown regions.

By far the most electrodes are located in white matter (40.3%) and unknown areas (12.6%). Unknown areas are contacts that were not able to be labelled through the Freesurfer parcellation, for example a contact located just outside of the cortex. Thereafter, electrodes are predominantly located in the superior temporal sulcus, hippocampus and the inferior parietal gyrus. See Fig. 3 for a full breakdown of anatomical regions and the number of electrodes implanted in those areas.

## Data Records

The SingleWordProductionDutch-iBIDS dataset^67^ is available at 10.17605/OSF.IO/NRGX6. The raw data files (XDF format) were converted to Neurodata Without Borders (NWB; https://www.nwb.org/) format and organised in the iBIDS^68^ data structure format using custom Python scripts. The NWB format allows for compact storage of multiple data streams within a single file. It is compatible to the iBIDS structure, a community-driven effort to improve the transparency, reusibility and reproducibility of iEEG data.

The data is structured following the BIDS version 1.7.0 specification (https://bids-specification.readthedocs.io/en/stable/). The root folder contains metadata of the participants (*participants.tsv*), subject specific data folders (i.e., *sub-01*) and a derivatives folder. The subject specific folders contain .tsv files with information about the implanted electrode coordinates (*_electrodes.tsv*), recording montage (*_channels.tsv*) and event markers (*_events.tsv*). The *_ieeg.nwb* file contains three raw data streams as timeseries (*iEEG*, *Audio* and *Stimulus*), which are located in the *acquisition* container. Descriptions of recording aspects and of specific .tsv columns are provided in correspondingly named .json files (i.e., *participants.json*). The derivatives folder contains the pial surface cortical meshes of the right (*_rh_pial.mat*) and left (*_lh_pial.mat*) hemisphere, the brain anatomy (*_brain.mgz*), the Destrieux atlas (*_aparc.a2009s* + *aseg.mgz*) and a white matter atlas (*_wmparc.mgz*) per subject, derived from the Freesurfer pipeline. The description column in the *_channels.tsv* file refers to the anatomical labels derived from the Destrieux atlas^66^.

The iBIDS dataset passed a validation check using the BIDS Validator (https://bids-standard.github.io/bids-validator/) and manual inspection of each datafile.

## Technical Validation

We validate the recorded data by demonstrating that a spectral representation of speech can be reconstructed from the neural recordings using a simple linear regression model. This analysis is similar to a previous analysis in ECoG^69^.

### Checking for acoustic contamination

Acoustic contamination of neural recordings has been reported by Roussel *et al*.^70^. To check the presented dataset for acoustic contamination in the neural timeseries, we apply the method provided by the authors and correlate spectral energy between audio and neural data. We do not find any significant correlations (*p* > 0.01) on the diagonal of the contamination matrix for any of the participants. The risk for falsely rejecting the hypothesis of no contamination is therefore smaller than 1%.

### Feature extraction

We extract the Hilbert envelope of the broadband high-frequency activity (70–170 Hz) for each contact using an IIR bandpass filter (filter order 4). To attenuate the first two harmonics of the 50 Hz line noise, we used two IIR bandstop filters (filter order 4). All filters were applied forward and backward so that no phase-shift is introduced. We averaged the envelope over 50 ms windows with a frameshift of 10 ms. To include temporal information into the decoding process, non-overlapping neighboring windows up to 200 ms into the past and future were stacked. Features are normalized to zero mean and unit variance using the mean and standard deviation of the training data. The same transform is then applied to the evaluation data.

The audio data is first downsampled to 16 kHz. To extract features for the audio data, we subsequently calculated the Short-Term-Fourier-Transform in windows of 50 ms with an frameshift of 10 ms. As the frameshift between neural and audio data is the same, there is a correspondence between audio and neural feature vectors. The resulting spectrogram is then compressed into a log-mel representation^71^ using 23 triangular filter banks.

### Decoding model

To reduce the dimensionality of our decoding problem, we compress the feature space to the first 50 principal components. Principal components are estimated for each fold individually on the training data. The first 50 principal components explain between 29% and 76% of the variance depending on the participant.

We reconstruct the log-mel spectrogram from the high-frequency features using linear regression models. In these models, the high-frequency feature vector is multiplied with a weight matrix to reconstruct the log-mel spectrogram. The weights are determined using a least-squares approach.

As a baseline, we chose 1000 random split points, at least 10% distant from the beginning and end of the data, and swapped the audio spectrogram on this split point. In this procedure, which is also called random circular shift, the temporal structure and auto-regressive properties of speech are maintained. We then correlated these spectrograms with the original spectrogram to estimate a distribution of chance correlation coefficients.

### Waveform reconstruction

The log-mel spectrogram does not contain the phase information anymore and an audio waveform can thus not be reconstructed directly. We utilize the method by Griffin and Lim^72^ for waveform reconstruction, in which the phase is initialized with noise and then iteratively modified. For a good algorithmic discription of the method, see^73^.

### Results

All results are obtained in a non-shuffled 10-fold cross validation in which 9 folds are used for training and the remaining fold is used for evaluation. This process is repeated until each fold has been used for evaluation exactly once.

#### Spectrograms can be reconstructed

**Fig. 4:**
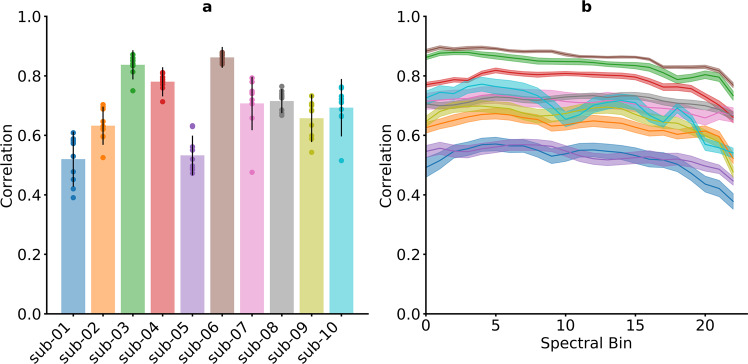
Results for the spectral reconstruction. (**a**) Mean correlation coefficients for each participant across all spectral bins and folds. Reconstruction of the spectrogram is possible for all 10 participants. Whiskers indicate standard deviations. Results of individual folds are illustrated by points. (**b**) Mean correlation coefficients for each spectral bin. Correlations are stable across all spectral bins. Shaded areas show standard errors.

**Fig. 5:**
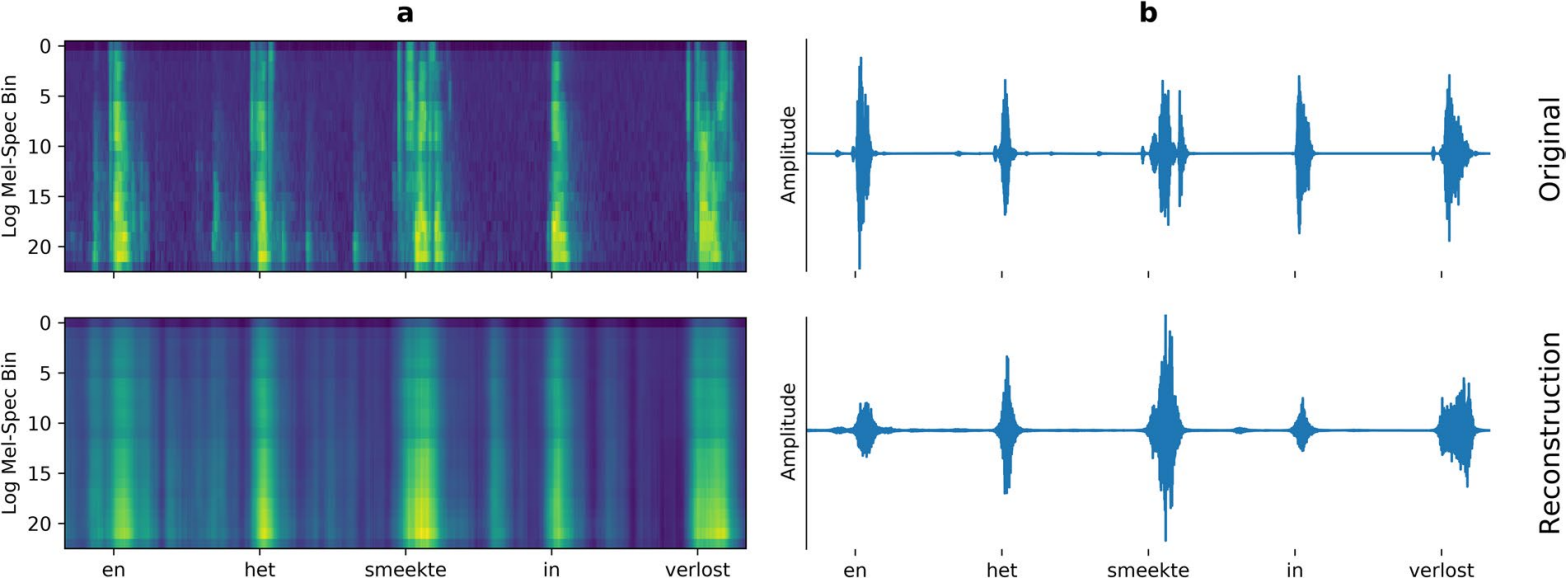
Spectrograms (**a**) and waveforms (**b**) of the original (top) and reconstructed (bottom) audio. The example contains five individual words from sub-06. While the linear regression approach captures speech and silent intervals very accurately, the finer spectral dynamics within speech are lost.

We evaluate the spectral reconstructions in terms of Pearson correlation coefficient between the spectral coefficients of the spectrogram of the original speech and the reconstructed spectrogram. For all 10 participants, speech spectrograms can be reconstructed from the neural data using linear regression (Fig. 4a) with higher correlations than all 1000 randomizations. Reconstruction results were consistent across all 23 mel-scaled spectral coefficients (Fig. 4b) and consistently above all randomization in all frequency ranges. Inspecting the spectrograms further (Fig. 5a), it can be seen that the results are mostly driven by the accurate reconstruction of speech versus silence. The spectral variations within speech are not captured by the linear regression approach. The Pearson correlation is not a perfect evaluation metric as this lack of detail during speech does not have a large impact on the score. We utilize the Pearson correlation here as a better metric has yet to be identified. By providing this open dataset, we hope that researchers developing more advanced metrics, such as the Spectro-Temporal Glimpsing Index (STGI)^74^ or the extended Short-Time Objective Intelligibility (eSTOI)^75^, will have the means to address this problem. Similarly, we hope that the dataset will be useful for developing and evaluating models that improve the quality of the reconstructed speech, such as those that are more informed about speech processes (e.g. Unit Selection^29^) or neural network approaches with enough trainable parameters to produce high-quality speech^26,27,76–78^.

#### Waveforms of speech can be reconstructed

Using the method by Griffin and Lim^72^, we can recreate an audio waveform from the reconstructed spectrograms (Fig. 5b). The timing between original and reconstructed waveforms is very similar, but listening to the audio shows that a substantial part of the audio quality is lost due to the synthesis approach. This is particularly clear when listening to the audio recreated from the original spectrogams (*_orig_synthesized.wav*). State-of-the-art synthesis approaches, such as WaveGlow^79^ or WaveNet^80^, may be applied to the current dataset to evaluate an improvement in the reconstruction quality.

## Usage Notes

### iBIDS data

Scripts to handle the data can be obtained from our Github repository: https://github.com/neuralinterfacinglab/SingleWordProductionDutch.

#### Data loading and feature extraction

Neural traces, synchronized audio and experimental markers can be loaded using the provided *extract_features.py* script. High-frequency features are subsequently extracted and aligned to logarithmic mel-scaled spectrograms. Electrode channel names are also loaded.

#### Spectrogram reconstruction & waveform synthesis

Reconstruction of the spectrogram as well as the resynthesis to an audio waveform is performed in the *reconstruction_minimal.py* script.

### Anatomical data

Electrode locations can be found in the participant folder (*_electrodes.tsv*) and then be visualized using the cortical meshes (*_lh_pial.mat* and *_rh_pial.mat*) within the derivatives folder. These mesh files contain vertices coordinates and triangles, which are described by indices corresponding to vertex numbers. The T1-weighted brain anatomy, the Destrieux parcellation and a white matter parcellation can also be found in the derivatives folder.

## Data Availability

All Python code to re-run the technical validation described in this report can be found on our Github: https://github.com/neuralinterfacinglab/SingleWordProductionDutch. The code relies on the numpy^81^, scipy^82^, pynwb^83^, scikit-learn^84^ and pandas^85^ packages.

## References

[1] Wolpaw J, Birbaumer N, McFarland D, Pfurtscheller G, Vaughan T (2002). Brain–computer interfaces for communication and control. Clinical neurophysiology.

[2] Rabbani Q, Milsap G, Crone NE (2019). The potential for a speech brain–computer interface using chronic electrocorticography. Neurotherapeutics.

[3] Schultz T (2017). Biosignal-based spoken communication: A survey. IEEE/ACM Transactions on Audio, Speech and Language Processing.

[4] Chakrabarti S, Sandberg HM, Brumberg JS, Krusienski DJ (2015). Progress in speech decoding from the electrocorticogram. Biomedical Engineering Letters.

[5] Herff, C. & Schultz, T. Automatic speech recognition from neural signals: a focused review. *Frontiers in neuroscience* **10** (2016). 10.3389/fnins.2016.00429 PMC5037201 27729844

[6] Bocquelet F, Hueber T, Girin L, Chabardès S, Yvert B (2016). Key considerations in designing a speech brain-computer interface. Journal of Physiology-Paris.

[7] Moses DA (2021). Neuroprosthesis for decoding speech in a paralyzed person with anarthria. New England Journal of Medicine.

[8] Tourville JA, Guenther FH (2011). The diva model: A neural theory of speech acquisition and production. Language and cognitive processes.

[9] Hickok G (2012). Computational neuroanatomy of speech production. Nature Reviews Neuroscience.

[10] Piai V (2016). Direct brain recordings reveal hippocampal rhythm underpinnings of language processing. Proceedings of the National Academy of Sciences.

[11] Duff MC, Brown-Schmidt S (2012). The hippocampus and the flexible use and processing of language. Frontiers in human neuroscience.

[12] Covington NV, Duff MC (2016). Expanding the language network: Direct contributions from the hippocampus. Trends in Cognitive Sciences.

[13] Hebb AO, Ojemann GA (2013). The thalamus and language revisited. Brain and Language.

[14] Klostermann F, Ehlen F (2013). Functional roles of the thalamus for language capacities. Frontiers in systems neuroscience.

[15] Brunner P (2009). A practical procedure for real-time functional mapping of eloquent cortex using electrocorticographic signals in humans. Epilepsy & Behavior.

[16] Mugler EM (2014). Direct classification of all american english phonemes using signals from functional speech motor cortex. Journal of neural engineering.

[17] Ramsey NF (2018). Decoding spoken phonemes from sensorimotor cortex with high-density ECoG grids. Neuroimage.

[18] Lotte, F. *et al*. Electrocorticographic representations of segmental features in continuous speech. *Frontiers in human neuroscience* **9** (2015). 10.3389/fnhum.2015.00097 PMC4338752 25759647

[19] Mugler EM (2018). Differential representation of articulatory gestures and phonemes in precentral and inferior frontal gyri. Journal of Neuroscience.

[20] Kellis, S. *et al*. Decoding spoken words using local field potentials recorded from the cortical surface. *Journal of Neural Engineering* **7** (2010). 10.1088/1741-2560/7/5/056007 PMC2970568 20811093

[21] Herff, C. *et al*. Brain-to-text: decoding spoken phrases from phone representations in the brain. *Frontiers in neuroscience* **9** (2015). 10.3389/fnins.2015.00217 PMC4464168 26124702

[22] Moses DA, Mesgarani N, Leonard MK, Chang EF (2016). Neural speech recognition: continuous phoneme decoding using spatiotemporal representations of human cortical activity. Journal of neural engineering.

[23] Moses DA, Leonard MK, Chang EF (2018). Real-time classification of auditory sentences using evoked cortical activity in humans. Journal of neural engineering.

[24] Makin, J. G., Moses, D. A. & Chang, E. F. Machine translation of cortical activity to text with an encoder–decoder framework. *Tech. Rep*., Nature Publishing Group (2020). 10.1038/s41593-020-0608-8 PMC10560395 32231340

[25] Milsap G (2019). Keyword spotting using human electrocorticographic recordings. Frontiers in neuroscience.

[26] Anumanchipalli GK, Chartier J, Chang EF (2019). Speech synthesis from neural decoding of spoken sentences. Nature.

[27] Angrick, M. *et al*. Speech synthesis from ECoG using densely connected 3D convolutional neural networks. *Journal of neural engineering* (2019). 10.1088/1741-2552/ab0c59 PMC6822609 30831567

[28] Wang, R., Wang, Y. & Flinker, A. Reconstructing speech stimuli from human auditory cortex activity using a wavenet approach. In *2018 IEEE Signal Processing in Medicine and Biology Symposium (SPMB)*, 1–6 (IEEE, 2018).

[29] Herff C (2019). Generating natural, intelligible speech from brain activity in motor, premotor, and inferior frontal cortices. Frontiers in Neuroscience.

[30] Martin, S. *et al*. Decoding spectrotemporal features of overt and covert speech from the human cortex. *Frontiers in neuroengineering* **7** (2014). 10.3389/fneng.2014.00014 PMC4034498 24904404

[31] Martin S (2016). Word pair classification during imagined speech using direct brain recordings. Scientific reports.

[32] Proix T (2022). Imagined speech can be decoded from low-and cross-frequency intracranial EEG features. Nature communications.

[33] Angrick M (2021). Real-time synthesis of imagined speech processes from minimally invasive recordings of neural activity. Communications biology.

[34] Meng, K. *et al*. Implementation of a closed-loop BCI system for real-time speech synthesis under clinical constraints. In *2022 10th International Winter Conference on Brain-Computer Interface (BCI)*, 1–6 (IEEE, 2022).

[35] Parvizi J, Kastner S (2018). Promises and limitations of human intracranial electroencephalography. Nature neuroscience.

[36] Stavisky, S. D. *et al*. Decoding speech from intracortical multielectrode arrays in dorsal “arm/hand areas” of human motor cortex. In *2018 40th Annual International Conference of the IEEE Engineering in Medicine and Biology Society (EMBC)*, 93–97 (IEEE, 2018). 10.1109/EMBC.2018.8512199 30440349

[37] Stavisky SD (2019). Neural ensemble dynamics in dorsal motor cortex during speech in people with paralysis. Elife.

[38] Stavisky SD (2020). Speech-related dorsal motor cortex activity does not interfere with iBCI cursor control. Journal of Neural Engineering.

[39] Wilson, G. H. *et al*. Decoding spoken english phonemes from intracortical electrode arrays in dorsal precentral gyrus. *bioRxiv* (2020). 10.1088/1741-2552/abbfef PMC8293867 33236720

[40] Bartels J (2008). Neurotrophic electrode: method of assembly and implantation into human motor speech cortex. Journal of neuroscience methods.

[41] Brumberg J, Wright E, Andreasen D, Guenther F, Kennedy P (2011). Classification of intended phoneme production from chronic intracortical microelectrode recordings in speech motor cortex. Frontiers in Neuroscience.

[42] Guenther FH (2009). A wireless brain-machine interface for real-time speech synthesis. PloS one.

[43] van der Loo LE (2017). Methodology, outcome, safety and *in vivo* accuracy in traditional frame-based stereoelectroencephalography. Acta neurochirurgica.

[44] Iida K, Otsubo H (2017). Stereoelectroencephalography: indication and efficacy. Neurologia medico-chirurgica.

[45] Herff C, Krusienski DJ, Kubben P (2020). The potential of stereotactic-EEG for brain-computer interfaces: Current progress and future directions. Frontiers in Neuroscience.

[46] Mamun K (2015). Movement decoding using neural synchronization and inter-hemispheric connectivity from deep brain local field potentials. Journal of neural engineering.

[47] Wandelt SK (2022). Decoding grasp and speech signals from the cortical grasp circuit in a tetraplegic human. Neuron.

[48] Wang L, Zhang X, Zhong X, Zhang Y (2013). Analysis and classification of speech imagery EEG for BCI. Biomedical signal processing and control.

[49] Sereshkeh AR, Trott R, Bricout A, Chau T (2017). Online EEG classification of covert speech for brain–computer interfacing. International journal of neural systems.

[50] Garca-Salinas JS, Villaseñor-Pineda L, Reyes-Garca CA, Torres-Garca AA (2019). Transfer learning in imagined speech EEG-based BCIs. Transfer learning in imagined speech EEG-based BCIs.

[51] Cooney, C., Folli, R. & Coyle, D. Mel frequency cepstral coefficients enhance imagined speech decoding accuracy from EEG. In *2018 29th Irish Signals and Systems Conference (ISSC)*, 1–7 (IEEE, 2018).

[52] Krishna, G., Tran, C., Carnahan, M. & Tewfik, A. Advancing speech recognition with no speech or with noisy speech. In *2019 27th European Signal Processing Conference (EUSIPCO)*, 1–5 (IEEE, 2019).

[53] Sharon RA, Narayanan SS, Sur M, Murthy AH (2020). Neural speech decoding during audition, imagination and production. IEEE Access.

[54] Islam, M. M. & Shuvo, M. M. H. DenseNet based speech imagery EEG signal classification using Gramian Angular Field. In *2019 5th International Conference on Advances in Electrical Engineering (ICAEE)*, 149–154, 10.1109/ICAEE48663.2019.8975572 (2019).

[55] Zhao, S. & Rudzicz, F. Classifying phonological categories in imagined and articulated speech. In *2015 IEEE International Conference on Acoustics, Speech and Signal Processing (ICASSP)*, 992–996 (IEEE, 2015).

[56] Wang, J., Kim, M., Hernandez-Mulero, A. W., Heitzman, D. & Ferrari, P. Towards decoding speech production from single-trial magnetoencephalography (MEG) signals. In *2017 IEEE International Conference on Acoustics, Speech and Signal Processing (ICASSP)*, 3036–3040 (IEEE, 2017).

[57] Dash, D., Ferrari, P. & Wang, J. Decoding imagined and spoken phrases from non-invasive neural (MEG) signals. *Frontiers in Neuroscience* (2020). 10.3389/fnins.2020.00290 PMC7154084 32317917

[58] Sereshkeh AR, Yousefi R, Wong AT, Chau T (2018). Online classification of imagined speech using functional near-infrared spectroscopy signals. Journal of neural engineering.

[59] Herff, C., Heger, D., Putze, F., Guan, C. & Schultz, T. Cross-subject classification of speaking modes using fNIRS. In *International Conference on Neural Information Processing*, 417–424 (Springer, 2012).

[60] Liu Y, Ayaz H (2018). Speech recognition via fNIRS based brain signals. Frontiers in neuroscience.

[61] Rezazadeh Sereshkeh A, Yousefi R, Wong AT, Rudzicz F, Chau T (2019). Development of a ternary hybrid fNIRS-EEG brain–computer interface based on imagined speech. Brain-Computer Interfaces.

[62] Van Son, R. J., Binnenpoorte, D., Heuvel, H. v. d. & Pols, L. The IFA corpus: a phonemically segmented dutch ”open source” speech database. In *7th European Conference on Speech Communication and Technology* (Aalborg, Denmark, 2001).

[63] McFee, B. *et al*. librosa: Audio and music signal analysis in python. In *Proceedings of the 14th python in science conference*, vol. 8, 18–25 (Citeseer, 2015).

[64] Kothe, C. Lab streaming layer (LSL). https://github.com/sccn/labstreaminglayer **26**, 2015 (2014).

[65] Hamilton LS, Chang DL, Lee MB, Chang EF (2017). Semi-automated anatomical labeling and inter-subject warping of high-density intracranial recording electrodes in electrocorticography. Frontiers in Neuroinformatics.

[66] Destrieux C, Fischl B, Dale A, Halgren E (2010). Automatic parcellation of human cortical gyri and sulci using standard anatomical nomenclature. NeuroImage.

[67] Herff C, Verwoert M (2022). Open Science Framework.

[68] Holdgraf C (2019). iEEG-BIDS, extending the brain imaging data structure specification to human intracranial electrophysiology. Scientific data.

[69] Herff, C. *et al*. Towards direct speech synthesis from ECoG: A pilot study. In *Engineering in Medicine and Biology Society (EMBC)*, 2016 *IEEE 38th Annual International Conference of the*, 1540–1543 (IEEE, 2016). 10.1109/EMBC.2016.7591004 28268620

[70] Roussel P (2020). Observation and assessment of acoustic contamination of electrophysiological brain signals during speech production and sound perception. Journal of Neural Engineering.

[71] Stevens SS, Volkmann J, Newman EB (1937). A scale for the measurement of the psychological magnitude pitch. The Journal of the Acoustical Society of America.

[72] Griffin D, Lim J (1984). Signal estimation from modified short-time fourier transform. IEEE Transactions on acoustics, speech, and signal processing.

[73] Bayram I (2012). An analytic wavelet transform with a flexible time-frequency covering. IEEE Transactions on Signal Processing.

[74] Edraki, A., Chan, W. Y., Jensen, J. & Fogerty, D. A spectro-temporal glimpsing index (STGI) for speech intelligibility prediction. In *22nd Annual Conference of the International Speech Communication Association, INTERSPEECH* 2021, 2738–2742 (International Speech Communication Association, 2021).

[75] Jensen J, Taal CH (2016). An algorithm for predicting the intelligibility of speech masked by modulated noise maskers. *IEEE/ACM Transactions on*. Audio, Speech, and Language Processing.

[76] Angrick, M. *et al*. Speech synthesis from stereotactic EEG using an electrode shaft dependent multi-input convolutional neural network approach. In *2021 43rd Annual International Conference of the IEEE Engineering in Medicine & Biology Society (EMBC)*, 6045–6048 (IEEE, 2021). 10.1109/EMBC46164.2021.9629711 34892495

[77] Kohler, J. *et al*. Synthesizing speech from intracranial depth electrodes using an encoder-decoder framework. *arXiv preprint arXiv:2111.01457* (2021).

[78] Wang, R. *et al*. Distributed feedforward and feedback processing across perisylvian cortex supports human speech. *bioRxiv* (2021).

[79] Prenger, R., Valle, R. & Catanzaro, B. Waveglow: A flow-based generative network for speech synthesis. In *ICASSP 2019-2019 IEEE International Conference on Acoustics, Speech and Signal Processing (ICASSP)*, 3617–3621 (IEEE, 2019).

[80] Van Den Oord, A. *et al*. Wavenet: A generative model for raw audio. *arXiv preprint arXiv:1609.03499* (2016).

[81] Harris CR (2020). Array programming with numpy. Nature.

[82] Virtanen P (2020). Scipy 1.0: fundamental algorithms for scientific computing in python. Nature methods.

[83] Teeters JL (2015). Neurodata without borders: creating a common data format for neurophysiology. Neuron.

[84] Pedregosa F (2011). Scikit-learn: Machine learning in python. the Journal of machine Learning research.

[85] McKinney W (2011). Pandas: a foundational python library for data analysis and statistics. Python for high performance and scientific computing.

